# Naloxegol hydrogen oxalate displaying a hydrogen-bonded layer structure

**DOI:** 10.1107/S2056989018003675

**Published:** 2018-03-09

**Authors:** Thomas Gelbrich, Christoph Langes, Marijan Stefinovic, Ulrich J. Griesser

**Affiliations:** a University of Innsbruck, Institute of Pharmacy, Innrain 52, 6020 Innsbruck, Austria; b Sandoz GmbH, Biochemiestrasse 10, 6250 Kundl, Austria

**Keywords:** crystal structure, hydrogen bonding, topology, oxalate, pharmaceuticals

## Abstract

In the salt (5α,6α)-6-[(2,5,8,11,14,17,20-hepta­oxadocosan-22-yl)­oxy]-3,14-dihy­droxy-17-(prop-2-en-1-yl)-4,5-ep­oxy­morphinan-17-ium hydrogen oxalate, C_34_H_54_NO_11_^+^·C_2_HO_4_^−^ the polyether unit of the naloxegol cation adopts the shape of a squashed open letter ‘O’. In the crystal, the hydrogen oxalate anions are linked into a chain by O—H⋯O hydrogen bonds. Each naloxegol unit is hydrogen bonded to three hydrogen oxalate ions *via* two O—H⋯O and one N—H⋯O inter­actions. The resulting hydrogen-bonded two-dimensional layer structure is 3,5-connected and has the **3,5 L50** topology.

## Abstract 2

Nodes representing hydrogen-bonded naloxegol and hemioxalate units form a 3,5-connected net which has the **3,5 L50** topology.

## Chemical context   

Naloxegol {(5α,6α)-17-allyl-6-[(20-hy­droxy-3,6,9,12,15,18-hexa­oxaicos-1-yl)­oxy]-4,5-ep­oxy­morphinan-3,14-diol} is a pegylated derivative of naloxone which serves as a peripherally acting *m*-opioid receptor antagonist. This compound was developed for the oral treatment of opioid-induced constipation in adults with chronic non-cancer pain, and is currently marketed under the trade name Movantik by AstraZeneca. Åslund *et al.* (2012 ▸) have described two forms, denoted as *A* and *B*, of naloxegol oxalate. Form *B* was reported as showing ‘a sharp endothermic peak at 92.5° C’ (365.5 K) in the DSC thermogram with a heat of fusion of Δ_fus_ *H* = 96.1 J g^−1^ (71.29 kJ mol^−1^). Herein we report the crystal structure of naloxegol hydrogen oxalate (I) (C_34_H_54_NO_11_^+^ C_2_HO_4_^−^), which is identical with form *B* described by Åslund *et al.* (2012 ▸). The unequivocal identity with form *B* is evidenced by the match of the X-ray powder diffraction data and the good agreement of the melting data [*T*_fus(onset)_ = 363.9 ± 0.3 K, *T*_fus(peak)_ = 366.7 ± 0.3 K, Δ_fus_ *H* = 70.4 ± 0.6 kJ mol^−1^] with those reported by Åslund *et al.* (2012 ▸).
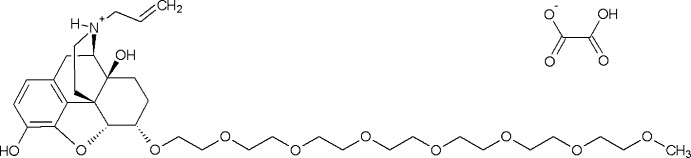


## Structural commentary   

**Figure 1:**
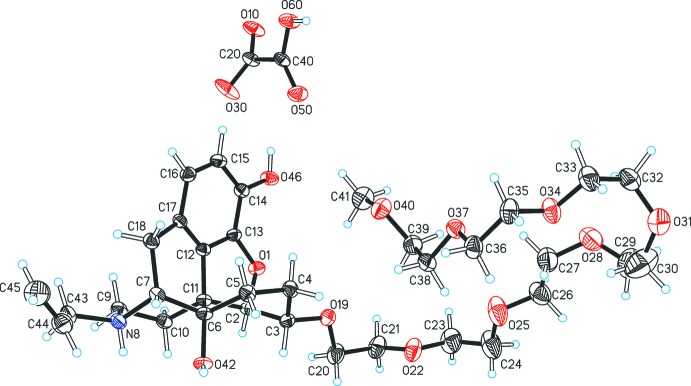
The asymmetric unit of (I), with displacement ellipsoids drawn at the 50% probability level and H atoms drawn as spheres of arbitrary size.

The geometry of the morphine scaffold in the title structure (I) is very similar to that of the parent mol­ecule in the naloxone hydro­chloride dihydrate structure (Klein *et al.*, 1987 ▸), except for the conformation of the cyclo­hexyl ring (C2–C6/C11) (Figs. 1 ▸ and 2 ▸). In (I), the puckering parameters for this ring of *q* = 85.3 (2)° and θ = 76.6 (3)° indicate a conformation between boat and twist boat (Cremer & Pople, 1975 ▸; Boeyens, 1978 ▸). The conformation of the 2-propenyl group at N8 is characterised by the torsion angle N8—C43—C44—C45 of 133.6 (4)°, which differs substanti­ally from the corresponding value, −96.2°, in the naloxone hydro­chloride dihydrate. The polyether unit adopts the shape of a squashed open letter O. Using the nomenclature for torsion angles recommended by Markley *et al.* (1998 ▸), the conformation of the fragment (C3, O19–C41) can be described by the sequence *tg* ^+^ *t tg* ^−^[[*t*]] *ttg* ^−^ *g* ^−^ *g* ^−^ *g* ^+^ *g* ^+^ *g* ^−^ *t tg* ^−^ *t tg* ^−^ *t*. All O—C—C—O angles are *gauche* except for O25—C26—C27—O28. Six consecutive *gauche*-type torsion angles are associated with a 180° turn within the chain section (C26–O34) (Fig. 1 ▸, Table 1 ▸). The hydrogen oxalate anion displays a twisted conformation with a torsion angle O1*O*—C2*O*—C4*O*—O5*O* of −143.3 (3)°.

## Supra­molecular features   

The naloxegol cation contains one NH group and two OH groups, which can serve as hydrogen-bond donor groups, and the hydrogen oxalate contains another OH group. Neighbouring hydrogen oxalate ions are hydrogen bonded (Table 2 ▸) to one another (O6*O*—H6*O*⋯O1*O* ^iii^), so that a chain structure parallel to the *b* axis is formed. Each naloxegol unit serves as a bridge between two such hydrogen oxalate chains in that it provides two bonds, O42—H42⋯O3*O* ^ii^ and N8—H8⋯O1*O* ^i^, to two different anions belonging to one hydrogen-bonded hydrogen oxalate chain, The third bond, O46—H46⋯O5*O*, connects to a second anion chain (Fig. 3 ▸). Altogether, each naloxegol cation forms three hydrogen bonds to three hydrogen oxalate ions, and each anion is engaged in five one-point hydrogen-bonding inter­actions with two hydrogen oxalate and three naloxegol units. The 3,5-connected layer structure (Fig. 4 ▸) resulting from these inter­actions lies in the *ab* plane. It possesses the **3,5L50** topology and has the point symbol (3.5^2^)(3^2^.5^3^.6^4^.7), wherein the naloxegol and hydrogen oxalate nodes are represented by the string (3.5^2^) and (3^2^.5^3^.6^4^.7), respectively.

## Database survey   

**Figure 2:**
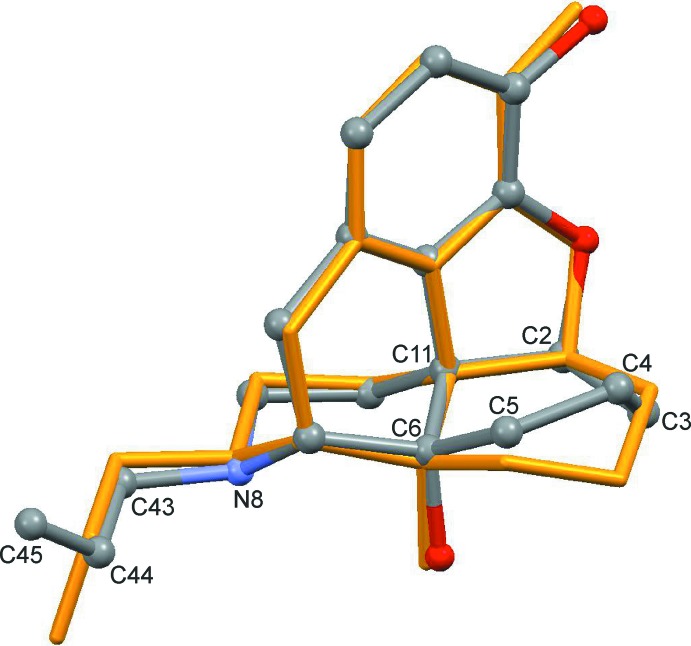
Overlay of the morphine scaffolds of (I) and naloxone hydro­chloride dihydrate (Klein *et al.*, 1987 ▸; coloured orange), obtained by least-squares fitting all ring atoms except for (C2–C6/C11).

**Figure 3:**
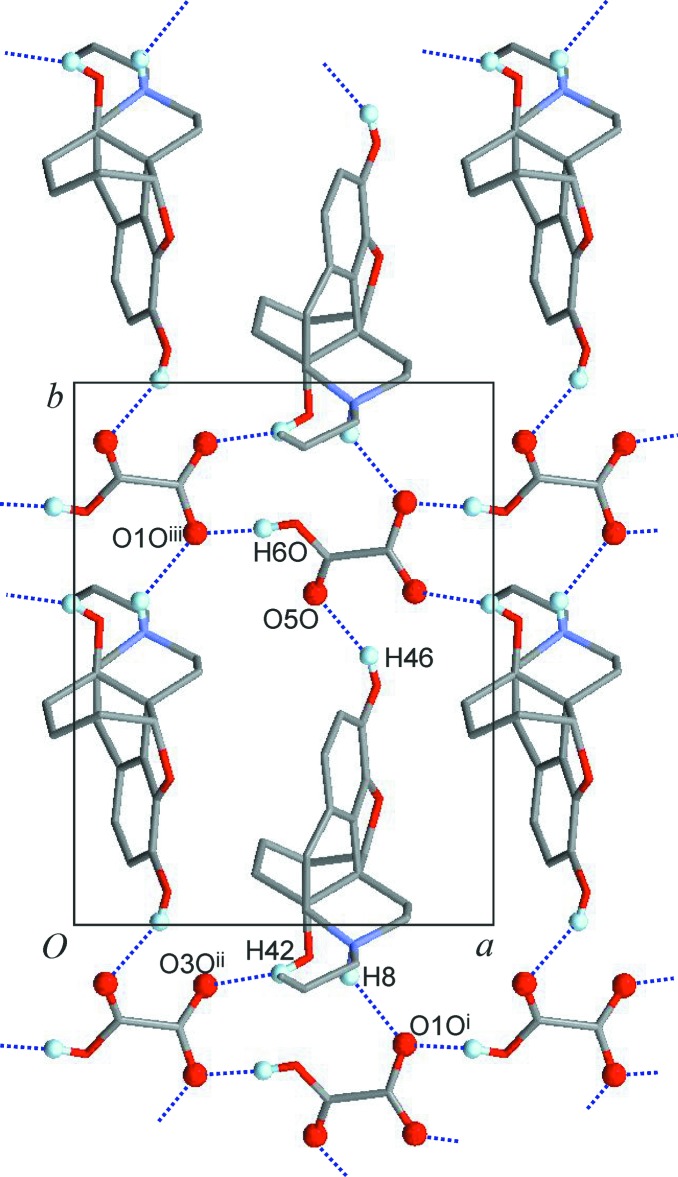
Hydrogen-bonded layer structure of (I), viewed along the *c* axis. H and O atoms directly engaged in hydrogen bonding are drawn as balls. All other H atoms and the polyether group are omitted for clarity.

**Figure 4:**
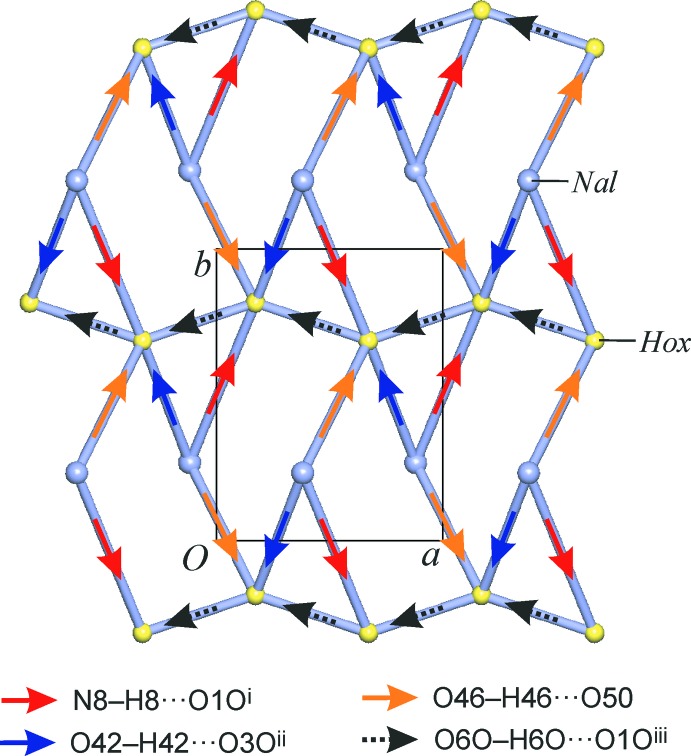
Topological representation in the manner proposed by Hursthouse *et al.* (2015 ▸) of the hydrogen-bonded layer structure with the **3,5 L50** topology (Nal = naloxegol, Hox = hydrogen oxalate). The net is viewed along the *c* axis. Note that the naloxegol nodes are placed at the centroid of the mol­ecule rather than the center of its morphine scaffold.

Crystal structures of a hydro­chloride dihydrate (Karle, 1974 ▸; Sime *et al.*, 1975 ▸; Klein *et al.*, 1987 ▸; see Fig. 2 ▸) and a hydro­chloride anhydrate (Sugimoto *et al.*, 2007 ▸) of the parent mol­ecule naloxone are known.

Heptaglyme (heptaethyleneglycol dimethyl ether) has been used as a multidentate ligand in Ba (FIXKAY; Wei *et al.*, 1987 ▸), Ca (RUFWUK; Arunasalam *et al.*, 1997 ▸) and Gd (YOMBUX; Baxter *et al.*, 1995 ▸) complexes. The hepta­glyme conformations in these crystals differ substanti­ally from the chain geometry found in (I). For example, the hepta­glyme complex with barium thio­cyanate displays a regular sequence *tg^+^t tg* ^−^ *t tg* ^+^ *t tg* ^−^ *t tg* ^+^ *t tg* ^−^ *tg* ^+^ *t* with sign alternation (Wei *et al.*, 1987 ▸).

## Synthesis and crystallization   

Naloxegol was obtained as a viscous transparent yellow oil (purity 95.05%). Approximately 4000 mg (6.14 mmol) of the free base were dissolved in 30 ml of ethyl­acetate and 774 mg (1 meq) of oxalic acid dihydrate (Merck) suspended in 20 ml of ethyl­acetate. The free-base solution was added dropwise to the suspended counter-ion. Stirring at room temperature for 15 minutes transformed the gel-like material into a suspension. The oxalate salt formation was complete after continued stirring for 12 h at ambient temperature. The slurry was then separated from the mother liquor by centrifuge and then dried *in* vacuo at ambient temperature (yield 3700 mg = 4.99 mmol = 81% of theory). The PXRD pattern of the dried product was found to match that of form *B* reported in Åslund *et al.* (2012 ▸).

A sample of form *B* (50 mg) was dissolved in 0.3 ml of 2-propanol under slight heating. Filtration through a syringe filter (pore size 0.44 microns) yielded a clear solution. The solution was allowed to cool to room temperature. Crystallization in a closed vial yielded single crystals suitable for a crystal structure determination. Typical crystal morphologies of (I) obtained by evaporation from different organic solvents are shown in Fig. S1 of the Supporting information.

## Refinement   

Crystal data, data collection and structure refinement details are summarized in Table 3 ▸. All H atoms were identified in difference maps. Methyl H atoms were idealized and included as rigid groups allowed to rotate but not tip and refined with *U*_iso_ set to 1.5*U*_eq_(C) of the parent carbon atom. All other H atoms bound to carbon atoms were positioned geometrically and refined with *U*_iso_ set to 1.2*U*_eq_(C) of the parent carbon atom. Hydrogen atoms in OH and NH groups were refined with restrained distances [O—H = 0.84 (1) Å; N—H = 0.88 (1) Å] and their *U*_iso_ parameters were refined freely. The absolute structure was established by anomalous-dispersion effects.

The largest residual peak of 0.73 e Å^−3^ is located 1.00 Å from C30. An alternative refinement of a disorder model with a split C30 position was attempted, but resulted in a few unreasonably short intra­molecular H⋯H distances for the minor disorder fragment. This feature could not be eliminated even with the aplication of a suitable anti-bumping restraint.

The topology of the hydrogen-bonded structures was determined and classified with the programs *ADS* and *IsoTest* of the *TOPOS* package (Blatov, 2006 ▸) in the manner described by Baburin & Blatov (2007 ▸).

## Supplementary Material

Crystal structure: contains datablock(s) I. DOI: 10.1107/S2056989018003675/fy2125sup1.cif

Structure factors: contains datablock(s) I. DOI: 10.1107/S2056989018003675/fy2125Isup2.hkl

Typical morphologies, hot-stage microscopy and DSC thermogram of naloxegol hydrogen oxalate. DOI: 10.1107/S2056989018003675/fy2125sup3.pdf

CCDC reference: 1827135

Additional supporting information:  crystallographic information; 3D view; checkCIF report

## Figures and Tables

**Table 1:** Selected torsion angles (°)

C3—O19—C20—C21	−179.4 (3)	C29—C30—O31—C32	60.2 (8)
O19—C20—C21—O22	69.0 (4)	C30—O31—C32—C33	80.4 (5)
C20—C21—O22—C23	−177.0 (3)	O31—C32—C33—O34	−74.8 (4)
C21—O22—C23—C24	−177.9 (4)	C32—C33—O34—C35	−178.3 (3)
O22—C23—C24—O25	−69.9 (6)	C36—C35—O34—C33	−177.8 (3)
C23—C24—O25—C26	−136.1 (5)	O34—C35—C36—O37	−68.8 (4)
C24—O25—C26—C27	−173.2 (5)	C35—C36—O37—C38	174.7 (3)
O25—C26—C27—O28	−177.6 (4)	C36—O37—C38—C39	−176.6 (3)
C26—C27—O28—C29	−78.4 (5)	O37—C38—C39—O40	−70.0 (3)
C27—O28—C29—C30	−81.2 (7)	C38—C39—O40—C41	−169.8 (3)
O28—C29—C30—O31	−70.5 (8)		

**Table 2:** Hydrogen-bond geometry (Å, °)

*D*—H⋯*A*	*D*—H	H⋯*A*	*D*⋯*A*	*D*—H⋯*A*
N8—H8⋯O1*O* ^i^	0.88 (1)	2.23 (3)	2.911 (3)	134 (3)
O42—H42⋯O3*O* ^ii^	0.84 (1)	2.12 (2)	2.906 (3)	157 (4)
O46—H46⋯O5*O*	0.84 (1)	2.09 (3)	2.853 (3)	151 (5)
O6*O*—H6*O*⋯O1*O* ^iii^	0.85 (1)	1.69 (2)	2.536 (3)	173 (6)

Symmetry codes: (i) 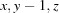; (ii) 
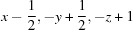
; (iii) 
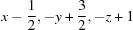
.

**Table 3:** Experimental details

Crystal data	
Chemical formula	C_34_H_54_NO_11_^+^·C_2_HO_4_^−^
*M*_r_	741.81
Crystal system, space group	Orthorhombic, *P*2_1_2_1_2_1_
Temperature (K)	173
*a*, *b*, *c* (Å)	10.3581 (1), 13.4039 (1), 26.1689 (2)
*V* (Å^3^)	3633.26 (5)
*Z*	4
Radiation type	Cu *K*α
μ (mm^−1^)	0.88
Crystal size (mm)	0.25 × 0.15 × 0.05

Data collection	
Diffractometer	Rigaku Oxford Diffraction Xcalibur Ruby Gemini Ultra
Absorption correction	Multi-scan (*CrysAlis PRO*; Rigaku OD, 2015 ▸)
*T*_min_, *T*_max_	0.809, 1.000
No. of measured, independent and observed [*I* > 2σ(*I*)] reflections	58082, 6563, 6429
*R*_int_	0.056
(sin θ/λ)_max_ (Å^−1^)	0.599

Refinement	
*R*[*F* ^2^ > 2σ(*F* ^2^)], *wR*(*F* ^2^), *S*	0.048, 0.126, 1.03
No. of reflections	6563
No. of parameters	488
No. of restraints	4
H-atom treatment	H atoms treated by a mixture of independent and constrained refinement
Δρ_max_, Δρ_min_ (e Å^−3^)	0.73, −0.43
Absolute structure	Flack *x* determined using 2777 quotients [(*I* ^+^)−(*I* ^−^)]/[(*I* ^+^)+(*I* ^−^)] (Parsons *et al.*, 2013 ▸)
Absolute structure parameter	0.00 (4)

Computer programs: *CrysAlis PRO* (Rigaku OD, 2015 ▸), *SHELXT* (Sheldrick, 2015*a* ▸), *SHELXL2014* (Sheldrick, 2015*b* ▸), *XP* in *SHELXTL* (Sheldrick, 2008 ▸), *Mercury* (Macrae *et al.*, 2006 ▸), *TOPOS* (Blatov, 2006 ▸), *PLATON* (Spek, 2009 ▸) and *publCIF* (Westrip, 2010 ▸).

## supplementary crystallographic information

### Crystal data

**Table:**

C_34_H_54_NO_11_^+^·C_2_HO_4_^−^	*D*_x_ = 1.356 Mg m^−^^3^
*M**_r_* = 741.81	Cu *K*α radiation, λ = 1.54184 Å
Orthorhombic, *P*2_1_2_1_2_1_	Cell parameters from 31215 reflections
*a* = 10.3581 (1) Å	θ = 3.4–67.5°
*b* = 13.4039 (1) Å	µ = 0.88 mm^−^^1^
*c* = 26.1689 (2) Å	*T* = 173 K
*V* = 3633.26 (5) Å^3^	Plate, colourless
*Z* = 4	0.25 × 0.15 × 0.05 mm
*F*(000) = 1592	

### Data collection

**Table:**

Riguaku Oxford Diffraction Xcalibur Ruby Gemini Ultra diffractometer	6563 independent reflections
Radiation source: fine-focus sealed X-ray tube, Enhance Ultra (Cu) X-ray Source	6429 reflections with *I* > 2σ(*I*)
Mirror monochromator	*R*_int_ = 0.056
Detector resolution: 10.3575 pixels mm^-1^	θ_max_ = 67.5°, θ_min_ = 3.4°
ω scans	*h* = −12→12
Absorption correction: multi-scan (CrysAlis PRO; Rigaku OD, 2015)	*k* = −16→16
*T*_min_ = 0.809, *T*_max_ = 1.000	*l* = −31→31
58082 measured reflections	

### Refinement

**Table:**

Refinement on *F*^2^	Secondary atom site location: difference Fourier map
Least-squares matrix: full	Hydrogen site location: mixed
*R*[*F*^2^ > 2σ(*F*^2^)] = 0.048	H atoms treated by a mixture of independent and constrained refinement
*wR*(*F*^2^) = 0.126	*w* = 1/[σ^2^(*F*_o_^2^) + (0.0807*P*)^2^ + 1.840*P*] where *P* = (*F*_o_^2^ + 2*F*_c_^2^)/3
*S* = 1.03	(Δ/σ)_max_ = 0.001
6563 reflections	Δρ_max_ = 0.73 e Å^−^^3^
488 parameters	Δρ_min_ = −0.43 e Å^−^^3^
4 restraints	Absolute structure: Flack *x* determined using 2777 quotients [(*I*^+^)-(*I*^-^)]/[(*I*^+^)+(*I*^-^)] (Parsons *et al.*, 2013)
Primary atom site location: structure-invariant direct methods	Absolute structure parameter: 0.00 (4)

### Special details

**Table:**

Geometry. All esds (except the esd in the dihedral angle between two l.s. planes) are estimated using the full covariance matrix. The cell esds are taken into account individually in the estimation of esds in distances, angles and torsion angles; correlations between esds in cell parameters are only used when they are defined by crystal symmetry. An approximate (isotropic) treatment of cell esds is used for estimating esds involving l.s. planes.

### Fractional atomic coordinates and isotropic or equivalent isotropic displacement parameters (Å^2^)

**Table:**

	*x*	*y*	*z*	*U*_iso_*/*U*_eq_	
O1	0.7271 (2)	0.23153 (15)	0.60003 (8)	0.0220 (4)	
C2	0.6886 (3)	0.1258 (2)	0.60078 (11)	0.0196 (6)	
H2	0.7575	0.0854	0.6177	0.024*	
C3	0.5616 (3)	0.1131 (2)	0.62988 (11)	0.0223 (6)	
H3	0.5480	0.0404	0.6366	0.017 (8)*	
C4	0.4487 (3)	0.1516 (2)	0.59856 (12)	0.0238 (6)	
H4A	0.4632	0.2229	0.5904	0.029*	
H4B	0.3688	0.1468	0.6192	0.029*	
C5	0.4304 (3)	0.0928 (2)	0.54844 (11)	0.0229 (6)	
H5A	0.3603	0.0436	0.5534	0.027*	
H5B	0.4025	0.1398	0.5214	0.027*	
C6	0.5516 (3)	0.0375 (2)	0.52989 (11)	0.0196 (6)	
C7	0.5514 (3)	0.0269 (2)	0.47054 (11)	0.0204 (6)	
H7	0.4709	−0.0094	0.4607	0.025*	
N8	0.6650 (2)	−0.03899 (18)	0.45687 (9)	0.0225 (5)	
H8	0.656 (4)	−0.0951 (17)	0.4738 (13)	0.028 (9)*	
C9	0.7908 (3)	0.0108 (2)	0.47010 (12)	0.0233 (6)	
H9A	0.8002	0.0730	0.4501	0.028*	
H9B	0.8635	−0.0340	0.4612	0.028*	
C10	0.7950 (3)	0.0347 (2)	0.52665 (11)	0.0218 (6)	
H10A	0.8735	0.0743	0.5341	0.026*	
H10B	0.8004	−0.0282	0.5463	0.026*	
C11	0.6759 (3)	0.0933 (2)	0.54401 (11)	0.0178 (5)	
C12	0.6642 (3)	0.1937 (2)	0.51828 (10)	0.0180 (5)	
C13	0.6953 (3)	0.2674 (2)	0.55230 (11)	0.0184 (5)	
C14	0.6840 (3)	0.3679 (2)	0.53902 (11)	0.0196 (5)	
C15	0.6339 (3)	0.3874 (2)	0.49073 (11)	0.0218 (6)	
H15	0.6254	0.4548	0.4801	0.026*	
C16	0.5954 (3)	0.3121 (2)	0.45718 (11)	0.0222 (6)	
H16	0.5598	0.3289	0.4248	0.025 (9)*	
C17	0.6093 (3)	0.2118 (2)	0.47115 (11)	0.0187 (5)	
C18	0.5532 (3)	0.1265 (2)	0.44095 (11)	0.0217 (6)	
H18A	0.6041	0.1178	0.4093	0.026*	
H18B	0.4637	0.1438	0.4309	0.026*	
O19	0.5624 (2)	0.16457 (16)	0.67753 (8)	0.0282 (5)	
C20	0.6280 (4)	0.1130 (3)	0.71702 (12)	0.0382 (8)	
H20A	0.7192	0.1023	0.7072	0.046*	
H20B	0.5875	0.0469	0.7225	0.046*	
C21	0.6215 (4)	0.1731 (3)	0.76544 (13)	0.0401 (8)	
H21A	0.6797	0.1434	0.7913	0.048*	
H21B	0.6513	0.2420	0.7586	0.048*	
O22	0.4955 (3)	0.1756 (2)	0.78457 (9)	0.0424 (6)	
C23	0.4912 (5)	0.2381 (4)	0.82948 (15)	0.0527 (11)	
H23A	0.5172	0.3068	0.8201	0.063*	
H23B	0.5536	0.2128	0.8550	0.063*	
C24	0.3631 (5)	0.2404 (5)	0.85200 (16)	0.0654 (15)	
H24A	0.3331	0.1710	0.8571	0.079*	
H24B	0.3687	0.2722	0.8861	0.079*	
O25	0.2718 (3)	0.2915 (3)	0.82276 (11)	0.0669 (11)	
C26	0.1924 (5)	0.3545 (3)	0.85016 (16)	0.0472 (10)	
H26A	0.2447	0.4096	0.8646	0.057*	
H26B	0.1535	0.3173	0.8790	0.057*	
C27	0.0876 (4)	0.3971 (4)	0.81770 (16)	0.0474 (9)	
H27A	0.1269	0.4316	0.7881	0.057*	
H27B	0.0337	0.3420	0.8044	0.057*	
O28	0.0081 (3)	0.4650 (2)	0.84424 (12)	0.0524 (7)	
C29	−0.0852 (6)	0.4273 (6)	0.8764 (2)	0.0780 (17)	
H29A	−0.1057	0.4793	0.9020	0.094*	
H29B	−0.0469	0.3703	0.8951	0.094*	
C30	−0.2056 (6)	0.3937 (5)	0.8542 (2)	0.0805 (18)	
H30A	−0.1831	0.3463	0.8267	0.097*	
H30B	−0.2506	0.3546	0.8809	0.097*	
O31	−0.2970 (3)	0.4601 (3)	0.83377 (12)	0.0574 (8)	
C32	−0.2612 (4)	0.5223 (3)	0.79260 (15)	0.0418 (8)	
H32A	−0.1729	0.5478	0.7987	0.050*	
H32B	−0.3202	0.5804	0.7916	0.050*	
C33	−0.2645 (4)	0.4709 (3)	0.74176 (15)	0.0407 (8)	
H33A	−0.3453	0.4321	0.7385	0.049*	
H33B	−0.2624	0.5211	0.7140	0.049*	
C35	−0.1524 (4)	0.3577 (3)	0.68942 (13)	0.0352 (8)	
H35A	−0.1437	0.4074	0.6616	0.042*	
H35B	−0.2330	0.3195	0.6838	0.042*	
O34	−0.1574 (2)	0.4068 (2)	0.73736 (9)	0.0376 (6)	
C36	−0.0393 (3)	0.2888 (3)	0.68912 (13)	0.0337 (7)	
H36A	−0.0422	0.2453	0.7197	0.040*	
H36B	−0.0427	0.2458	0.6584	0.040*	
O37	0.0775 (2)	0.34491 (17)	0.68908 (9)	0.0305 (5)	
C38	0.1863 (4)	0.2810 (3)	0.69353 (13)	0.0339 (7)	
H38A	0.1900	0.2360	0.6636	0.041*	
H38B	0.1773	0.2393	0.7246	0.041*	
C39	0.3081 (4)	0.3401 (3)	0.69666 (13)	0.0348 (8)	
H39A	0.2995	0.3922	0.7233	0.042*	
H39B	0.3806	0.2958	0.7063	0.042*	
O40	0.3342 (2)	0.3856 (2)	0.64859 (9)	0.0360 (5)	
C41	0.4599 (4)	0.4277 (3)	0.64774 (16)	0.0426 (9)	
H41A	0.4677	0.4773	0.6751	0.064*	
H41B	0.4748	0.4600	0.6147	0.064*	
H41C	0.5241	0.3749	0.6529	0.064*	
O42	0.5589 (2)	−0.06069 (15)	0.55118 (8)	0.0240 (4)	
H42	0.490 (2)	−0.091 (3)	0.5438 (15)	0.035 (11)*	
C43	0.6689 (3)	−0.0737 (2)	0.40195 (12)	0.0297 (7)	
H43A	0.6894	−0.0160	0.3798	0.036*	
H43B	0.7394	−0.1230	0.3981	0.036*	
C44	0.5460 (4)	−0.1197 (3)	0.38398 (14)	0.0366 (8)	
H44	0.5046	−0.1676	0.4052	0.044*	
C45	0.4926 (5)	−0.0967 (3)	0.34005 (16)	0.0479 (10)	
H45A	0.5324	−0.0490	0.3183	0.058*	
H45B	0.4141	−0.1277	0.3300	0.058*	
O46	0.7208 (2)	0.44043 (15)	0.57269 (8)	0.0261 (5)	
H46	0.702 (5)	0.495 (2)	0.5584 (16)	0.048 (13)*	
O1O	0.7871 (2)	0.77819 (16)	0.49246 (11)	0.0347 (6)	
C2O	0.7474 (3)	0.6898 (2)	0.49807 (13)	0.0236 (6)	
O3O	0.8110 (2)	0.61333 (17)	0.49334 (13)	0.0470 (7)	
C4O	0.6050 (3)	0.6764 (2)	0.51321 (12)	0.0216 (6)	
O5O	0.5729 (2)	0.61010 (17)	0.54246 (9)	0.0322 (5)	
O6O	0.5275 (2)	0.73960 (17)	0.49151 (10)	0.0315 (5)	
H6O	0.449 (2)	0.730 (4)	0.498 (2)	0.069 (16)*	

### Atomic displacement parameters (Å^2^)

**Table:**

	*U*^11^	*U*^22^	*U*^33^	*U*^12^	*U*^13^	*U*^23^
O1	0.0265 (10)	0.0180 (10)	0.0216 (9)	−0.0045 (8)	−0.0056 (8)	0.0002 (8)
C2	0.0226 (13)	0.0147 (13)	0.0215 (13)	0.0003 (11)	−0.0028 (11)	0.0019 (10)
C3	0.0278 (15)	0.0193 (13)	0.0198 (14)	−0.0014 (12)	0.0013 (11)	0.0016 (10)
C4	0.0199 (13)	0.0241 (14)	0.0273 (14)	0.0014 (12)	0.0030 (12)	−0.0002 (12)
C5	0.0173 (13)	0.0243 (14)	0.0271 (14)	0.0003 (11)	0.0003 (11)	−0.0005 (12)
C6	0.0189 (13)	0.0150 (12)	0.0249 (14)	−0.0013 (11)	−0.0005 (11)	0.0032 (11)
C7	0.0197 (13)	0.0178 (13)	0.0238 (14)	0.0019 (11)	−0.0010 (11)	−0.0014 (11)
N8	0.0252 (12)	0.0179 (12)	0.0243 (12)	0.0017 (10)	−0.0004 (10)	−0.0020 (10)
C9	0.0182 (13)	0.0226 (13)	0.0291 (15)	0.0027 (11)	0.0023 (11)	0.0004 (12)
C10	0.0189 (13)	0.0203 (13)	0.0262 (14)	0.0019 (11)	−0.0016 (11)	0.0007 (11)
C11	0.0184 (13)	0.0135 (12)	0.0215 (13)	−0.0007 (10)	−0.0008 (10)	0.0023 (10)
C12	0.0163 (12)	0.0164 (12)	0.0212 (13)	0.0012 (10)	0.0001 (10)	0.0024 (10)
C13	0.0176 (12)	0.0161 (13)	0.0217 (13)	−0.0008 (10)	0.0012 (10)	0.0041 (10)
C14	0.0177 (12)	0.0169 (13)	0.0244 (14)	−0.0025 (10)	0.0000 (11)	−0.0005 (11)
C15	0.0211 (13)	0.0165 (12)	0.0277 (14)	0.0007 (11)	0.0005 (11)	0.0037 (11)
C16	0.0219 (13)	0.0228 (14)	0.0219 (14)	0.0014 (11)	−0.0008 (11)	0.0023 (11)
C17	0.0186 (12)	0.0156 (12)	0.0218 (13)	0.0022 (10)	0.0025 (11)	−0.0001 (11)
C18	0.0240 (13)	0.0198 (13)	0.0214 (13)	0.0010 (11)	−0.0028 (11)	−0.0021 (11)
O19	0.0373 (13)	0.0283 (11)	0.0191 (10)	0.0036 (10)	0.0002 (9)	0.0000 (8)
C20	0.041 (2)	0.051 (2)	0.0220 (16)	0.0108 (17)	−0.0020 (14)	0.0020 (15)
C21	0.0387 (19)	0.058 (2)	0.0235 (16)	0.0002 (17)	−0.0030 (14)	−0.0006 (16)
O22	0.0419 (14)	0.0598 (17)	0.0256 (11)	0.0039 (13)	0.0025 (10)	−0.0024 (11)
C23	0.060 (3)	0.065 (3)	0.0329 (18)	0.004 (2)	−0.0070 (18)	−0.0092 (19)
C24	0.074 (3)	0.090 (4)	0.032 (2)	0.042 (3)	−0.008 (2)	−0.009 (2)
O25	0.071 (2)	0.102 (3)	0.0276 (13)	0.047 (2)	−0.0056 (14)	−0.0071 (15)
C26	0.058 (2)	0.044 (2)	0.039 (2)	0.0104 (19)	−0.0067 (18)	−0.0113 (17)
C27	0.047 (2)	0.056 (2)	0.039 (2)	0.0106 (19)	0.0011 (17)	−0.0036 (18)
O28	0.0580 (17)	0.0458 (16)	0.0534 (17)	0.0114 (14)	−0.0022 (14)	−0.0053 (13)
C29	0.070 (3)	0.105 (5)	0.059 (3)	0.026 (3)	0.016 (3)	0.020 (3)
C30	0.074 (4)	0.100 (5)	0.067 (3)	−0.013 (3)	−0.008 (3)	0.031 (3)
O31	0.0538 (18)	0.0617 (19)	0.0568 (18)	0.0099 (15)	0.0151 (14)	0.0050 (15)
C32	0.048 (2)	0.0321 (18)	0.045 (2)	0.0065 (17)	0.0026 (17)	−0.0059 (15)
C33	0.0324 (18)	0.049 (2)	0.0411 (19)	0.0085 (17)	−0.0011 (15)	−0.0056 (16)
C35	0.0342 (18)	0.047 (2)	0.0248 (16)	−0.0012 (16)	−0.0008 (13)	−0.0086 (14)
O34	0.0338 (12)	0.0496 (14)	0.0295 (12)	0.0102 (11)	−0.0037 (10)	−0.0114 (11)
C36	0.0366 (18)	0.0351 (18)	0.0294 (16)	−0.0035 (15)	0.0030 (14)	−0.0080 (14)
O37	0.0283 (11)	0.0313 (12)	0.0318 (12)	0.0049 (10)	−0.0001 (9)	−0.0001 (9)
C38	0.0380 (18)	0.0323 (17)	0.0315 (16)	0.0122 (15)	0.0023 (14)	0.0029 (13)
C39	0.0365 (18)	0.0407 (19)	0.0273 (16)	0.0099 (15)	−0.0043 (14)	0.0012 (14)
O40	0.0288 (12)	0.0489 (14)	0.0304 (12)	−0.0003 (11)	−0.0034 (9)	0.0052 (11)
C41	0.0294 (17)	0.055 (2)	0.044 (2)	−0.0014 (17)	−0.0054 (15)	0.0014 (17)
O42	0.0264 (11)	0.0151 (9)	0.0304 (11)	−0.0044 (8)	−0.0008 (9)	0.0043 (8)
C43	0.0410 (18)	0.0231 (15)	0.0248 (15)	0.0055 (14)	0.0005 (14)	−0.0067 (12)
C44	0.051 (2)	0.0250 (16)	0.0336 (17)	−0.0065 (15)	−0.0033 (16)	−0.0062 (13)
C45	0.057 (2)	0.044 (2)	0.043 (2)	−0.0039 (19)	−0.0128 (19)	−0.0030 (17)
O46	0.0336 (12)	0.0146 (10)	0.0302 (11)	−0.0038 (9)	−0.0051 (9)	−0.0006 (8)
O1O	0.0209 (10)	0.0170 (10)	0.0662 (16)	−0.0015 (8)	0.0043 (10)	0.0062 (10)
C2O	0.0180 (13)	0.0163 (13)	0.0364 (16)	0.0010 (11)	−0.0023 (12)	0.0000 (12)
O3O	0.0223 (11)	0.0216 (11)	0.097 (2)	0.0050 (9)	0.0067 (13)	−0.0051 (13)
C4O	0.0189 (13)	0.0140 (12)	0.0319 (15)	−0.0007 (10)	−0.0010 (11)	−0.0021 (11)
O5O	0.0284 (11)	0.0259 (11)	0.0422 (13)	−0.0028 (9)	0.0019 (10)	0.0087 (10)
O6O	0.0174 (10)	0.0288 (11)	0.0482 (14)	0.0057 (9)	−0.0007 (10)	0.0111 (10)

### Geometric parameters (Å, º)

**Table:**

O1—C13	1.378 (3)	C24—H24B	0.9900
O1—C2	1.473 (3)	O25—C26	1.380 (5)
C2—C3	1.529 (4)	C26—C27	1.492 (6)
C2—C11	1.553 (4)	C26—H26A	0.9900
C2—H2	1.0000	C26—H26B	0.9900
C3—O19	1.425 (4)	C27—O28	1.411 (5)
C3—C4	1.519 (4)	C27—H27A	0.9900
C3—H3	1.0000	C27—H27B	0.9900
C4—C5	1.542 (4)	O28—C29	1.377 (7)
C4—H4A	0.9900	C29—C30	1.447 (9)
C4—H4B	0.9900	C29—H29A	0.9900
C5—C6	1.537 (4)	C29—H29B	0.9900
C5—H5A	0.9900	C30—O31	1.405 (7)
C5—H5B	0.9900	C30—H30A	0.9900
C6—O42	1.431 (3)	C30—H30B	0.9900
C6—C11	1.534 (4)	O31—C32	1.412 (5)
C6—C7	1.560 (4)	C32—C33	1.499 (5)
C7—N8	1.514 (4)	C32—H32A	0.9900
C7—C18	1.543 (4)	C32—H32B	0.9900
C7—H7	1.0000	C33—O34	1.408 (4)
N8—C9	1.505 (4)	C33—H33A	0.9900
N8—C43	1.512 (4)	C33—H33B	0.9900
N8—H8	0.879 (14)	C35—O34	1.418 (4)
C9—C10	1.515 (4)	C35—C36	1.492 (5)
C9—H9A	0.9900	C35—H35A	0.9900
C9—H9B	0.9900	C35—H35B	0.9900
C10—C11	1.531 (4)	C36—O37	1.425 (4)
C10—H10A	0.9900	C36—H36A	0.9900
C10—H10B	0.9900	C36—H36B	0.9900
C11—C12	1.509 (4)	O37—C38	1.420 (4)
C12—C13	1.368 (4)	C38—C39	1.492 (5)
C12—C17	1.379 (4)	C38—H38A	0.9900
C13—C14	1.396 (4)	C38—H38B	0.9900
C14—O46	1.367 (3)	C39—O40	1.424 (4)
C14—C15	1.391 (4)	C39—H39A	0.9900
C15—C16	1.396 (4)	C39—H39B	0.9900
C15—H15	0.9500	O40—C41	1.420 (5)
C16—C17	1.400 (4)	C41—H41A	0.9800
C16—H16	0.9500	C41—H41B	0.9800
C17—C18	1.506 (4)	C41—H41C	0.9800
C18—H18A	0.9900	O42—H42	0.839 (14)
C18—H18B	0.9900	C43—C44	1.491 (5)
O19—C20	1.417 (4)	C43—H43A	0.9900
C20—C21	1.503 (5)	C43—H43B	0.9900
C20—H20A	0.9900	C44—C45	1.312 (6)
C20—H20B	0.9900	C44—H44	0.9500
C21—O22	1.398 (5)	C45—H45A	0.9500
C21—H21A	0.9900	C45—H45B	0.9500
C21—H21B	0.9900	O46—H46	0.842 (14)
O22—C23	1.444 (5)	O1O—C2O	1.263 (4)
C23—C24	1.452 (7)	C2O—O3O	1.225 (4)
C23—H23A	0.9900	C2O—C4O	1.538 (4)
C23—H23B	0.9900	C4O—O5O	1.219 (4)
C24—O25	1.396 (5)	C4O—O6O	1.298 (4)
C24—H24A	0.9900	O6O—H6O	0.848 (14)
			
C13—O1—C2	106.4 (2)	O22—C23—H23B	109.3
O1—C2—C3	110.3 (2)	C24—C23—H23B	109.3
O1—C2—C11	106.2 (2)	H23A—C23—H23B	107.9
C3—C2—C11	111.9 (2)	O25—C24—C23	114.0 (4)
O1—C2—H2	109.5	O25—C24—H24A	108.7
C3—C2—H2	109.5	C23—C24—H24A	108.7
C11—C2—H2	109.5	O25—C24—H24B	108.7
O19—C3—C4	108.1 (2)	C23—C24—H24B	108.7
O19—C3—C2	112.2 (2)	H24A—C24—H24B	107.6
C4—C3—C2	110.8 (2)	C26—O25—C24	114.8 (3)
O19—C3—H3	108.5	O25—C26—C27	111.8 (3)
C4—C3—H3	108.5	O25—C26—H26A	109.3
C2—C3—H3	108.5	C27—C26—H26A	109.3
C3—C4—C5	112.3 (2)	O25—C26—H26B	109.3
C3—C4—H4A	109.1	C27—C26—H26B	109.3
C5—C4—H4A	109.1	H26A—C26—H26B	107.9
C3—C4—H4B	109.1	O28—C27—C26	113.1 (3)
C5—C4—H4B	109.1	O28—C27—H27A	109.0
H4A—C4—H4B	107.9	C26—C27—H27A	109.0
C6—C5—C4	114.5 (2)	O28—C27—H27B	109.0
C6—C5—H5A	108.6	C26—C27—H27B	109.0
C4—C5—H5A	108.6	H27A—C27—H27B	107.8
C6—C5—H5B	108.6	C29—O28—C27	118.3 (4)
C4—C5—H5B	108.6	O28—C29—C30	118.3 (5)
H5A—C5—H5B	107.6	O28—C29—H29A	107.7
O42—C6—C11	108.1 (2)	C30—C29—H29A	107.7
O42—C6—C5	111.3 (2)	O28—C29—H29B	107.7
C11—C6—C5	112.0 (2)	C30—C29—H29B	107.7
O42—C6—C7	107.7 (2)	H29A—C29—H29B	107.1
C11—C6—C7	106.6 (2)	O31—C30—C29	122.4 (6)
C5—C6—C7	110.9 (2)	O31—C30—H30A	106.7
N8—C7—C18	112.2 (2)	C29—C30—H30A	106.7
N8—C7—C6	106.7 (2)	O31—C30—H30B	106.7
C18—C7—C6	114.9 (2)	C29—C30—H30B	106.7
N8—C7—H7	107.6	H30A—C30—H30B	106.6
C18—C7—H7	107.6	C30—O31—C32	119.2 (4)
C6—C7—H7	107.6	O31—C32—C33	113.6 (3)
C9—N8—C43	109.4 (2)	O31—C32—H32A	108.8
C9—N8—C7	111.1 (2)	C33—C32—H32A	108.8
C43—N8—C7	115.2 (2)	O31—C32—H32B	108.8
C9—N8—H8	111 (3)	C33—C32—H32B	108.8
C43—N8—H8	103 (3)	H32A—C32—H32B	107.7
C7—N8—H8	107 (3)	O34—C33—C32	109.6 (3)
N8—C9—C10	110.1 (2)	O34—C33—H33A	109.8
N8—C9—H9A	109.6	C32—C33—H33A	109.8
C10—C9—H9A	109.6	O34—C33—H33B	109.8
N8—C9—H9B	109.6	C32—C33—H33B	109.8
C10—C9—H9B	109.6	H33A—C33—H33B	108.2
H9A—C9—H9B	108.2	O34—C35—C36	108.7 (3)
C9—C10—C11	112.1 (2)	O34—C35—H35A	110.0
C9—C10—H10A	109.2	C36—C35—H35A	110.0
C11—C10—H10A	109.2	O34—C35—H35B	110.0
C9—C10—H10B	109.2	C36—C35—H35B	110.0
C11—C10—H10B	109.2	H35A—C35—H35B	108.3
H10A—C10—H10B	107.9	C33—O34—C35	112.6 (3)
C12—C11—C10	112.9 (2)	O37—C36—C35	109.9 (3)
C12—C11—C6	105.0 (2)	O37—C36—H36A	109.7
C10—C11—C6	110.7 (2)	C35—C36—H36A	109.7
C12—C11—C2	100.6 (2)	O37—C36—H36B	109.7
C10—C11—C2	111.1 (2)	C35—C36—H36B	109.7
C6—C11—C2	115.9 (2)	H36A—C36—H36B	108.2
C13—C12—C17	123.5 (3)	C38—O37—C36	110.8 (3)
C13—C12—C11	109.5 (2)	O37—C38—C39	110.8 (3)
C17—C12—C11	126.1 (2)	O37—C38—H38A	109.5
C12—C13—O1	113.2 (2)	C39—C38—H38A	109.5
C12—C13—C14	121.0 (3)	O37—C38—H38B	109.5
O1—C13—C14	125.6 (3)	C39—C38—H38B	109.5
O46—C14—C15	123.8 (2)	H38A—C38—H38B	108.1
O46—C14—C13	120.2 (3)	O40—C39—C38	109.9 (3)
C15—C14—C13	116.0 (3)	O40—C39—H39A	109.7
C14—C15—C16	122.8 (3)	C38—C39—H39A	109.7
C14—C15—H15	118.6	O40—C39—H39B	109.7
C16—C15—H15	118.6	C38—C39—H39B	109.7
C15—C16—C17	120.0 (3)	H39A—C39—H39B	108.2
C15—C16—H16	120.0	C41—O40—C39	111.0 (3)
C17—C16—H16	120.0	O40—C41—H41A	109.5
C12—C17—C16	116.4 (3)	O40—C41—H41B	109.5
C12—C17—C18	119.7 (2)	H41A—C41—H41B	109.5
C16—C17—C18	123.5 (3)	O40—C41—H41C	109.5
C17—C18—C7	113.4 (2)	H41A—C41—H41C	109.5
C17—C18—H18A	108.9	H41B—C41—H41C	109.5
C7—C18—H18A	108.9	C6—O42—H42	108 (3)
C17—C18—H18B	108.9	C44—C43—N8	113.9 (3)
C7—C18—H18B	108.9	C44—C43—H43A	108.8
H18A—C18—H18B	107.7	N8—C43—H43A	108.8
C20—O19—C3	113.8 (2)	C44—C43—H43B	108.8
O19—C20—C21	109.4 (3)	N8—C43—H43B	108.8
O19—C20—H20A	109.8	H43A—C43—H43B	107.7
C21—C20—H20A	109.8	C45—C44—C43	122.6 (4)
O19—C20—H20B	109.8	C45—C44—H44	118.7
C21—C20—H20B	109.8	C43—C44—H44	118.7
H20A—C20—H20B	108.2	C44—C45—H45A	120.0
O22—C21—C20	110.9 (3)	C44—C45—H45B	120.0
O22—C21—H21A	109.5	H45A—C45—H45B	120.0
C20—C21—H21A	109.5	C14—O46—H46	106 (3)
O22—C21—H21B	109.5	O3O—C2O—O1O	126.7 (3)
C20—C21—H21B	109.5	O3O—C2O—C4O	116.4 (3)
H21A—C21—H21B	108.1	O1O—C2O—C4O	116.9 (2)
C21—O22—C23	109.6 (3)	O5O—C4O—O6O	125.6 (3)
O22—C23—C24	111.8 (4)	O5O—C4O—C2O	120.5 (3)
O22—C23—H23A	109.3	O6O—C4O—C2O	113.8 (3)
C24—C23—H23A	109.3	C4O—O6O—H6O	114 (4)
			
C13—O1—C2—C3	−102.0 (2)	C17—C12—C13—C14	−6.1 (4)
C13—O1—C2—C11	19.4 (3)	C11—C12—C13—C14	−175.8 (3)
O1—C2—C3—O19	−47.5 (3)	C2—O1—C13—C12	−12.2 (3)
C11—C2—C3—O19	−165.5 (2)	C2—O1—C13—C14	162.7 (3)
O1—C2—C3—C4	73.5 (3)	C12—C13—C14—O46	−177.2 (3)
C11—C2—C3—C4	−44.5 (3)	O1—C13—C14—O46	8.3 (4)
O19—C3—C4—C5	−173.9 (2)	C12—C13—C14—C15	3.0 (4)
C2—C3—C4—C5	62.7 (3)	O1—C13—C14—C15	−171.5 (3)
C3—C4—C5—C6	−22.0 (3)	O46—C14—C15—C16	−179.2 (3)
C4—C5—C6—O42	88.8 (3)	C13—C14—C15—C16	0.6 (4)
C4—C5—C6—C11	−32.4 (3)	C14—C15—C16—C17	−1.4 (4)
C4—C5—C6—C7	−151.3 (2)	C13—C12—C17—C16	5.0 (4)
O42—C6—C7—N8	−51.1 (3)	C11—C12—C17—C16	173.0 (3)
C11—C6—C7—N8	64.7 (3)	C13—C12—C17—C18	−167.3 (3)
C5—C6—C7—N8	−173.2 (2)	C11—C12—C17—C18	0.6 (4)
O42—C6—C7—C18	−176.1 (2)	C15—C16—C17—C12	−1.3 (4)
C11—C6—C7—C18	−60.3 (3)	C15—C16—C17—C18	170.8 (3)
C5—C6—C7—C18	61.9 (3)	C12—C17—C18—C7	6.4 (4)
C18—C7—N8—C9	61.4 (3)	C16—C17—C18—C7	−165.4 (3)
C6—C7—N8—C9	−65.2 (3)	N8—C7—C18—C17	−97.9 (3)
C18—C7—N8—C43	−63.7 (3)	C6—C7—C18—C17	24.1 (3)
C6—C7—N8—C43	169.7 (2)	C4—C3—O19—C20	157.5 (3)
C43—N8—C9—C10	−173.1 (2)	C2—C3—O19—C20	−80.0 (3)
C7—N8—C9—C10	58.6 (3)	C3—O19—C20—C21	−179.4 (3)
N8—C9—C10—C11	−52.4 (3)	O19—C20—C21—O22	69.0 (4)
C9—C10—C11—C12	−62.4 (3)	C20—C21—O22—C23	−177.0 (3)
C9—C10—C11—C6	55.1 (3)	C21—O22—C23—C24	−177.9 (4)
C9—C10—C11—C2	−174.6 (2)	O22—C23—C24—O25	−69.9 (6)
O42—C6—C11—C12	177.3 (2)	C23—C24—O25—C26	−136.1 (5)
C5—C6—C11—C12	−59.7 (3)	C24—O25—C26—C27	−173.2 (5)
C7—C6—C11—C12	61.8 (3)	O25—C26—C27—O28	−177.6 (4)
O42—C6—C11—C10	55.1 (3)	C26—C27—O28—C29	−78.4 (5)
C5—C6—C11—C10	178.1 (2)	C27—O28—C29—C30	−81.2 (7)
C7—C6—C11—C10	−60.4 (3)	O28—C29—C30—O31	−70.5 (8)
O42—C6—C11—C2	−72.6 (3)	C29—C30—O31—C32	60.2 (8)
C5—C6—C11—C2	50.4 (3)	C30—O31—C32—C33	80.4 (5)
C7—C6—C11—C2	171.8 (2)	O31—C32—C33—O34	−74.8 (4)
O1—C2—C11—C12	−18.8 (3)	C32—C33—O34—C35	−178.3 (3)
C3—C2—C11—C12	101.6 (3)	C36—C35—O34—C33	−177.8 (3)
O1—C2—C11—C10	101.0 (3)	O34—C35—C36—O37	−68.8 (4)
C3—C2—C11—C10	−138.6 (2)	C35—C36—O37—C38	174.7 (3)
O1—C2—C11—C6	−131.4 (2)	C36—O37—C38—C39	−176.6 (3)
C3—C2—C11—C6	−11.0 (3)	O37—C38—C39—O40	−70.0 (3)
C10—C11—C12—C13	−106.3 (3)	C38—C39—O40—C41	−169.8 (3)
C6—C11—C12—C13	132.9 (2)	C9—N8—C43—C44	−177.1 (3)
C2—C11—C12—C13	12.2 (3)	C7—N8—C43—C44	−51.1 (3)
C10—C11—C12—C17	84.4 (3)	N8—C43—C44—C45	133.6 (4)
C6—C11—C12—C17	−36.4 (4)	O3O—C2O—C4O—O5O	35.8 (5)
C2—C11—C12—C17	−157.2 (3)	O1O—C2O—C4O—O5O	−143.3 (3)
C17—C12—C13—O1	169.0 (2)	O3O—C2O—C4O—O6O	−143.1 (3)
C11—C12—C13—O1	−0.7 (3)	O1O—C2O—C4O—O6O	37.8 (4)

### Hydrogen-bond geometry (Å, º)

**Table:**

*D*—H···*A*	*D*—H	H···*A*	*D*···*A*	*D*—H···*A*
N8—H8···O1*O*^i^	0.88 (1)	2.23 (3)	2.911 (3)	134 (3)
O42—H42···O3*O*^ii^	0.84 (1)	2.12 (2)	2.906 (3)	157 (4)
O46—H46···O5*O*	0.84 (1)	2.09 (3)	2.853 (3)	151 (5)
O6*O*—H6*O*···O1*O*^iii^	0.85 (1)	1.69 (2)	2.536 (3)	173 (6)

Symmetry codes: (i) *x*, *y*−1, *z*; (ii) *x*−1/2, −*y*+1/2, −*z*+1; (iii) *x*−1/2, −*y*+3/2, −*z*+1.
