# Supplementary material for: Naloxegol hydrogen oxalate displaying a hydrogen-bonded layer structure
Source: Acta Crystallogr E Crystallogr Commun. 2018 Mar 9;74(Pt 4):474–7. doi: 10.1107/S2056989018003675 (PMC5946971; doi:10.1107/S2056989018003675)
Supplement: Supplementary file 3 [file e-74-00474-sup3.pdf]

supporting information

Naloxegol hydrogen oxalate displaying an hydrogen-bonded layer structure

Thomas Gelbrich,[a]* Christoph Langes,[a] Marijan Stefinovic[b] and Ulrich J. Griesser[a]

[a]University of Innsbruck, Institute of Pharmacy, Innrain 52, 6020 Innsbruck, Austria, and [b]Sandoz GmbH, Biochemiestrasse 10, 6250 Kundl, Austria

Correspondence e-mail: thomas.gelbrich@uibk.ac.at

Typical morphologies resulting from the evaporation of naloxegol hydrogen oxalate solutions in organic solvents

Evaporation experiments of solutions of naloxegol hydrogen oxalate in different solvents resulted in either platy single crystals, druses or spherolites (Fig. S1a–f). The spherolites often grow slowly and incomplete from a small number of nucleation centres emerging in an amorphous (liquid) film of the substance, which remains after the solvent has largely evaporated (Fig. S1e–f).

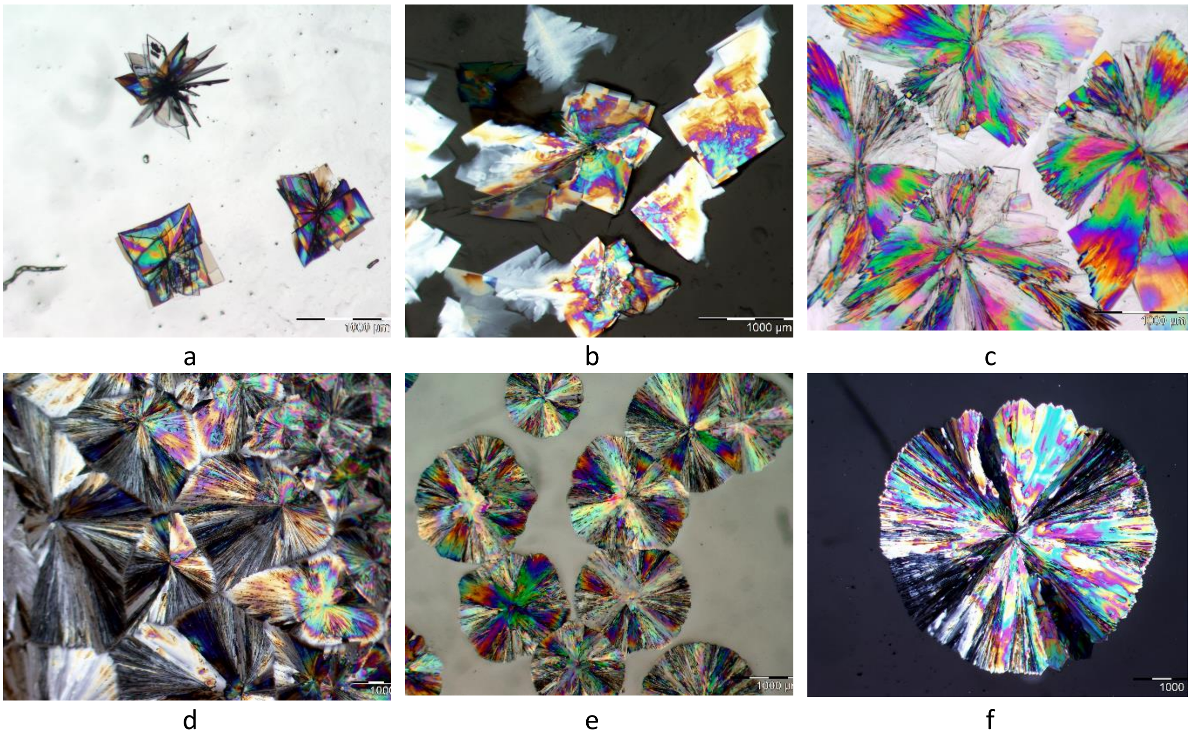

Fig. S1 Microscopic images of crystallization products of naloxegol hydrogen oxalate (form B) obtained by evaporation from different organic solvents: a) from butylacetate (druses of platy crystals), b) methyl isobutyl ketone, c) methylene chloride, d) 2-propanol, e) THF, f) ethanol.

Hot-stage microscopy

Experimental: An Olympus BH2 polarisation microscope (Olympus Optical GmbH, Vienna, Austria) equipped with a Kofler hot stage (Reichert Thermovar, Vienna, Austria) was used and images were recorded with an Olympus DP71 digital camera.

Result: On heating naloxegol oxalate recrystallized from ethyl acetate (platy crystals) no changes were observed below the melting range. The lack of condensation droplets or sublimates close to this range

indicates that the substance exhibits a very low vapour pressure. Melting occurs in a rather sluggish manner in the temperature range from 88 to 94 °C. On cooling melt preparations which still contain unmelted crystals of naloxegol oxalate (form B) the crystals grow very slow to play rectangles and squares (Fig. S2). Without seeds the melt does not recrystallize even on annealing the melt for about 20 hours between 60 and 80 °C (range showing the highest growth rate of crystals in the melt) indicating that primary nucleation does not occur in the melt.

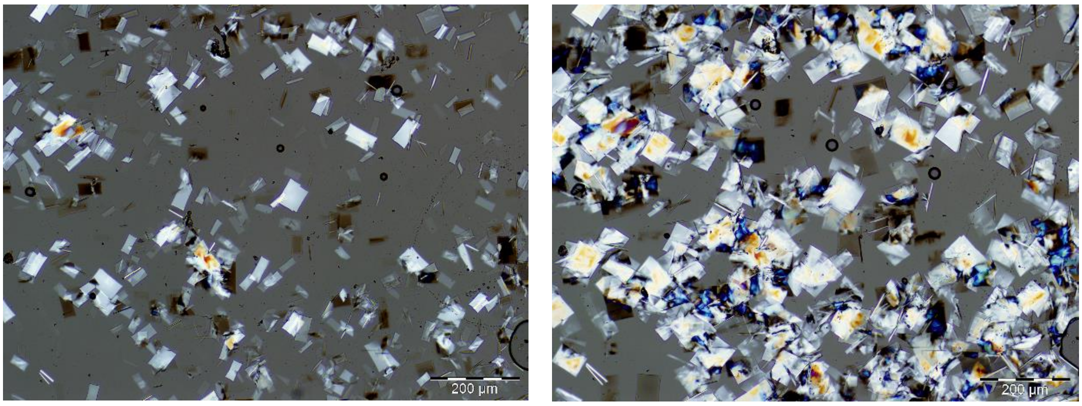

Fig. S2 Crystals of naloxegol oxalate growing in the seeded melt by annealing between 60 and 80 °C.

Differential Scanning Calorimetry (DSC)

Experimental: DSC thermograms were recorded with a DSC 7 (Perkin-Elmer, Norwalk, Ct., USA), operated with the Pyris 7.0 software. A few milligrams of the sample were accurately weighed (± 0.0005 mg) using a UM3 ultramicrobalance (Mettler, Greifensee, Switzerland) and heated in sealed 25 µl Al pans with a rate of 5 K min^{-1} under a dry nitrogen gas purge of 20 mL min^{-1}. The instrument was calibrated for temperature with pure benzophenone (m.p. 48.0 °C) and caffeine (m.p. 236.2 °C), and the energy calibration was performed with pure indium (m.p. 156.6 °C, heat of fusion 28.45 Jg^{-1}). The stated temperatures and enthalpies were determined from five measurements (mean with standard deviation).

Result: Fig. S3 shows a representative DSC curve of naloxegol oxalate (form B). The thermogram exhibits only a single melting endotherm with an onset temperature of 90.9 ± 0.3 °C (peak maximum of 93.7 ± 0.3 °C) and an enthalpy of fusion of 70.4 ± 0.6 kJ mol^{-1}. Thermogravimetric analysis (not shown) does not indicate any mass loss, confirming the absence of entrapped solvent(s) and the lack of sublimation or thermal decomposition.

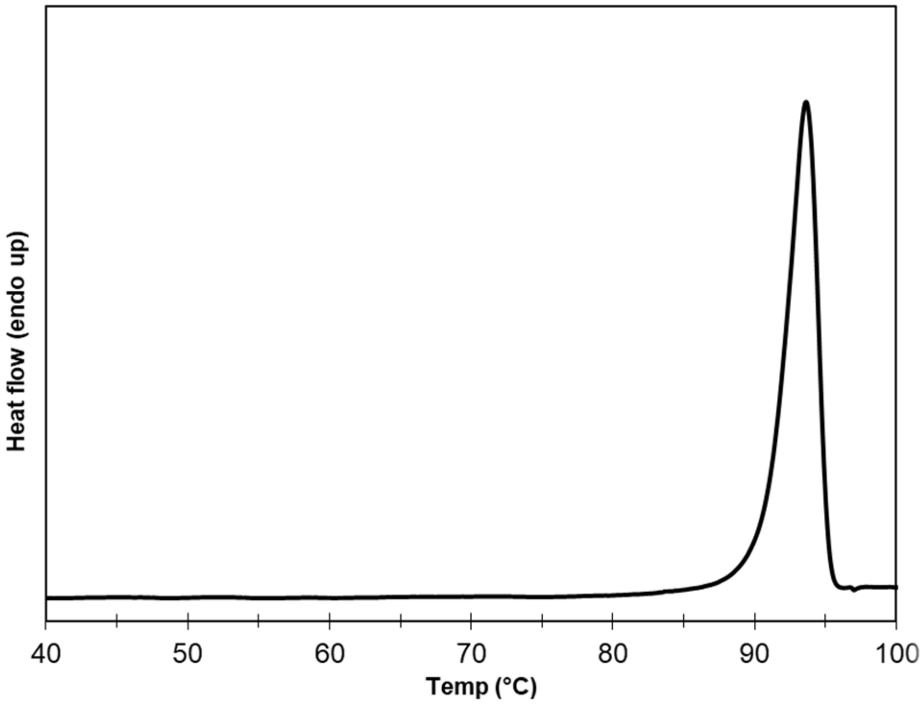

Fig. S3 DSC thermogram of naloxegol hydrogen oxalate showing a single melting endotherm.
